# Phenethyl Isothiocyanate (PEITC) Inhibits the Growth of Human Oral Squamous Carcinoma HSC-3 Cells through *G*_0_/*G*_1_ Phase Arrest and Mitochondria-Mediated Apoptotic Cell Death

**DOI:** 10.1155/2012/718320

**Published:** 2012-07-10

**Authors:** Po-Yuan Chen, Kai-Chun Lin, Jing-Pin Lin, Nou-Ying Tang, Jai-Sing Yang, Kung-Wen Lu, Jing-Gung Chung

**Affiliations:** ^1^Department of Biological Science and Technology, China Medical University, Taichung 40402, Taiwan; ^2^School of Pharmacy, China Medical University, Taichung 40402, Taiwan; ^3^School of Chinese Medicine, China Medical University, Taichung 40402, Taiwan; ^4^Department of Pharmacology, China Medical University, Taichung 40402, Taiwan; ^5^School of Post-Baccalaureate Chinese Medicine, China Medical University, Taichung 40402, Taiwan; ^6^Department of Biotechnology, Asia University, Taichung 413, Taiwan

## Abstract

Phenethyl isothiocyanate (PEITC), an effective anticancer and chemopreventive agent, has been reported to inhibit cancer cell growth through cell-cycle arrest and induction of apoptotic events in various human cancer cells models. However, whether PEITC inhibits human oral squamous cell carcinoma HSC-3 cell growth and its underlying mechanisms is still not well elucidated. In the present study, we evaluated the inhibitory effects of PEITC in HSC-3 cells and examined PEITC-modulated cell-cycle arrest and apoptosis. The contrast-phase and flow cytometric assays were used for examining cell morphological changes and viability, respectively. The changes of cell-cycle and apoptosis-associated protein levels were determined utilizing Western blotting in HSC-3 cells after exposure to PEITC. Our results indicated that PEITC effectively inhibited the HSC-3 cells' growth and caused apoptosis. PEITC induced *G*_0_/*G*_1_ phase arrest through the effects of associated protein such as p53, p21, p17, CDK2 and cyclin E, and it triggered apoptosis through promotion of Bax and Bid expression and reduction of Bcl-2, leading to decrease the levels of mitochondrial membrane potential (ΔΨ_*m*_), and followed the releases of cytochrome *c*, AIF and Endo G then for causing apoptosis in HSC-3 cells. These results suggest that PEITC could be an antitumor compound for oral cancer therapy.

## 1. Introduction

 The major contributors for oral cancer include tobacco and alcohol consumption [1, 2] as well as diets low in carotenoids, vitamin A, poor oral hygiene, and indoor air pollution [3–5]. In Taiwan, oral cancer patients are highly associated with betel quid chewing, cigarette smoking, and alcohol consumption [6]; about 7.9 individuals per 100,000 died of oral cancer in 2011, and it is the fifth most frequent cause of cancer death in Taiwan based upon the reports of the Cancer Registration System (Department of Health, Executive Yuan, Taiwan, R.O.C.). Approximately 90% of oral squamous cell carcinoma (OSCC) patients chew quid, and there is a relatively low incidence of oral malignancy in females. Surgery, radiotherapy, and chemotherapy are recognized to be the conventional treatments for patients with oral cancer [7], but the cure rates are not satisfactory.

Mitochondria play critical roles in apoptotic cell death [8], and it is becoming one of the principal targets in screening therapeutic agents against cancer [9–11]. After stimulation, the mitochondrial dysfunction (decreased the levels of mitochondrial membrane potential, ΔΨ_*m*_) which released cytochrome *c* and thereafter complexes with apoptotic protease activating factor-1 (Apaf-1) to form apoptosome activates caspase-9 and caspase-3, leading to apoptosis [11, 12]. Thus, many studies also focused to find compounds which can affect mitochondria for anticancer agents [11–14].

Phenethyl isothiocyanate (PEITC) presents in cruciferous vegetables which have been shown to decrease the risk of various types of malignancies [13, 14]. PEITC suppresses 4-(methylnitrosamino)-1-(3-pyridyl)-1-butone-induced pulmonary neoplasia in A/J mouse lung [14], exhibits cancer chemopreventive activity in rat [15], and reduces azoxymethane-induced colonic aberrant crypt foci formation [16]. PEITC induces apoptosis in human colon cancer HT-29 cells [17], prostate cancer cells [18], and osteogenic sarcoma U-2 OS cells [19]. Recently, in our laboratory, we also found that PEITC inhibits cell migration and invasion of colon cancer HT-29 cells [20] and human gastric cancer AGS cells [21]. However, there is no report to show that PEITC induced cytotoxic effects in human oral cancer cells. Our study investigated the cytotoxic effects of PEITC in human oral cancer HSC-3 cells *in vitro* and results indicated that PEITC induced cell death through the *G*_0_/*G*_1_ phase arrest and induction of apoptosis.

## 2. Materials and Methods

### 2.1. Chemicals and Reagents

 Phenethyl isothiocyanate (PEITC), dimethyl sulfoxide (DMSO), propidium iodide (PI), RNase A and Triton X-100 were obtained from Sigma-Aldrich Corp. (St. Louis, MO, USA). DMEM/F-12 (1 : 1), penicillin-streptomycin, trypsin-EDTA, fetal bovine serum (FBS) and L-glutamine were obtained from Gibco/Life Technologies (Carlsbad, CA, USA). 4′,6′-diamidino-2-phenylindole hydrochloride (DAPI), 2′,7′-dichlorodihydrofluorescein diacetate (DCFH-DA), glycine, N-[4-[6-[(acetyloxy)methoxy]-2,7-dichloro-3-oxo-3H-xanthen-9-yl]-2-[2-[2-[bis [2-[(acetyloxy)methoxy]-2-oxyethyl]amino]-5-methylphenoxy]ethoxy]phenyl]-N-[2-[(acetyloxy)methoxy]-2-oxyethyl]-, (acetyloxy)methyl ester (Fluo-3/AM) and 3,3′-dihexyloxacarbocyanine iodide (DiOC_6_) were purchased from Molecular Probes/Life Technologies (Eugene, OR, USA).

### 2.2. Cell Culture

The human oral squamous cell carcinoma HSC-3 cell line was kindly provided by Professor Pei-Jung Lu (National Cheng Kung University). Cells were maintained in DMEM/F-12 (1 : 1) containing 10% FBS, 2 mM L-glutamine, 100 Units/mL penicillin and 100 *μ*g/mL streptomycin at 37°C in a humidified atmosphere of 5% CO_2_.

### 2.3. Morphological Changes Observation and Cell Viability

 HSC-3 cells (2 × 10^5^) were placed in 12-well plates, and then PEITC was added to each well at final concentrations of 0.5, 1, 2, 2.5 and 5 *μ*M. DMSO at 0.5% in media served as a vehicle control. Cells were incubated for 24, 48 and 72 h. For morphological changes, cells in each well were examined for 24 and 48 h and photographed under a phase-contrast microscope [22, 23]. Cell viability was determined by a PI exclusion and flow cytometric procedure as previously described [22]. The data was presented from three separate experiments.

### 2.4. Analysis for Cell Cycle Distribution

HSC-3 cells (2 × 10^5^) were placed in 12-well plates, and then PEITC was added to each well at final concentrations of 0.5, 1, 2, 2.5 and 5 *μ*M for 24 and 48 h. DMSO at 0.5% in media served as a vehicle control. Then cells from each well were harvested individually by centrifugation and were measured the cell cycle distribution and sub-G_1_ phase determination by flow cytometric assay. Briefly, isolated cells were fixed gently by 70% ethanol at 4°C overnight and then re-suspended in PBS containing 40 *μ*g/mL PI and 0.1 mg/mL RNase A and 0.1% Triton X-100 in dark room for 30 min at 37°C, then were analyzed with a flow cytometer equipped with an argon ion laser at 488 nm wavelength as described previously [22, 23]. The results were done for three independent experiments.

### 2.5. DAPI Staining

HSC-3 cells (2 × 10^5^) in 12-well plates were treated with PEITC at final concentrations of 0.5, 1, 2, 2.5 and 5 *μ*M for 24 and 48 h and 0.5% DMSO as a vehicle control. Cells in each well were then individually stained by DAPI and photographed under fluorescence microscopy as described elsewhere [24, 25]. The level of each image was assayed in three independent experiments with similar results.

### 2.6. Flow Cytometric Assays for Reactive Oxygen Species (ROS) Production, Levels of ΔΨ_*m*_ and Cytosolic Ca^2+^

HSC-3 cells (2 × 10^5^ per well) placed in 12-well plates were treated with 2.5 *μ*M PEITC for 3, 6, 9, 12, 24 and 48 h to determine the level of ROS, ΔΨ_*m*_ and the cytosolic Ca^2+^. Cells were harvested and suspended in 500 *μ*L of DCFH-DA (10 *μ*M) for ROS measurement, in 500 *μ*L of Fluo-3/AM (2.5 *μ*g/mL) for Ca^2+^ levels examination and in 500 *μ*L of DiOC_6_ (1 *μ*mol/L) for ΔΨ_*m*_ determinations. Finally, all samples were incubated at 37°C for 30 min before being analyzed by flow cytometry as described previously [26, 27]. These results were carried out for three independent experiments.

### 2.7. Western Blotting for Protein Levels Analysis

HSC-3 cells (1 × 10^7^ per dish) were placed in 75-T flask and were treated with 2.5 *μ*M PEITC then incubated for 0, 6, 12, 24, 48 and 72 h. Cells from each treatments were harvested and lysed with ice-cold lysis buffer (PRO-PREPTM protein extraction solution, iNtRON Biotechnology, Seongnam-si, Gyeonggi-do, Korea) [23, 28]. Then all samples were determined the total protein. Proteins in each treatment were separated by SDS-PAGE, transferred to Immobilon-P transfer membrane (Millipore, Bedford, MA, USA), blocked with 5% (w/v) nonfat milk, probed with primary antibodies [p15, cdc25A, CDK6, cycin D, p53, p27, p21, CDK2, cyclin E, Fas, FasL, caspase-8, Bcl-2, Bid, xIAP, cytochrome *c*, caspase-9 and -3 (Santa Cruz Biotechnology, CA, USA), AIF, Endo G, NF-*κ*B p65, GRP78 and caspase-4 (R&D Systems, Minneapolis, MN, USA), Bax (Cat. 04-434), and Endo G (Cat. AB3639) (Merck Millipore, Billerica, MA, USA)]. They were then detected using the ECL kit (Millipore) and autoradiography using X-ray film [23, 28]. Each membrane was stripped and reported with anti-*β*-actin antibody for ensuring that equal proteins were loaded [23, 28]. The results were done for three independent experiments.

### 2.8. Docking and Score Using Accelrys Software and Ligand-Fit

The score functions in the Discovery Studio 2.5 which we used to predict the protein active binding pockets and simulate the possible ligand poses to present the interaction forces between the cdc25A (protein) and PEITC (ligand). There are two major parts of the LigandFit [29] docking: specify the region of the receptor to use as the binding site for docking. Site partitioning maybe applied to select parts of the binding site during docking, and dock ligands to the specified site. This part consists of the following steps: conformational search to generate candidate ligand conformations for docking, compare the ligand shape and protein-binding site shape by computing their size of possession, minimize the rigid body energy of candidate ligand pose/conformation by using the Dockscore calculation. All the simulations were also applied by the Forcefield of the Chemistry at CHARMM (Chemistry at Harvard Macromolecular Mechanics). CHARMM was parameterized by experimental data. It has been used widely for simulations ranging from small molecules to solvated complexes of large biological macromolecules. CHARMM performs well over a broad range of calculations and simulations, including minima, time-dependent dynamic behavior, and barriers to rotation, vibrational frequencies, and free energy. CHARMM uses a flexible and comprehensive energy function as follow:

(1) E(pot)=Ebond+Etorsion+Eoop+Eelect.+EvdW+Econstraint+Euser,

where the out-of-plane (OOP) angle is an important torsion. The van der Waals term is derived from rare-gas potentials, and the electrostatic term can be scaled to mimic solvent effects. Hydrogen-bond energy is not included as a separate term as in AMBER [30]. Instead, hydrogen-bond energy is implicit in the combination of van der Waals and electrostatic terms. The data was presented from three separate analyses with similar results.

### 2.9. Statistical Analysis

The results are presented as mean ± S.E.M, and the difference between the PEITC-treated and control groups was analyzed by Student's *t*-test, a probability of *P* < 0.05 being considered significant.

## 3. Results

### 3.1. PEITC Induced Cell-Morphological Changes and Decreased the Percentage of Viable Cells

**Figure 1:**
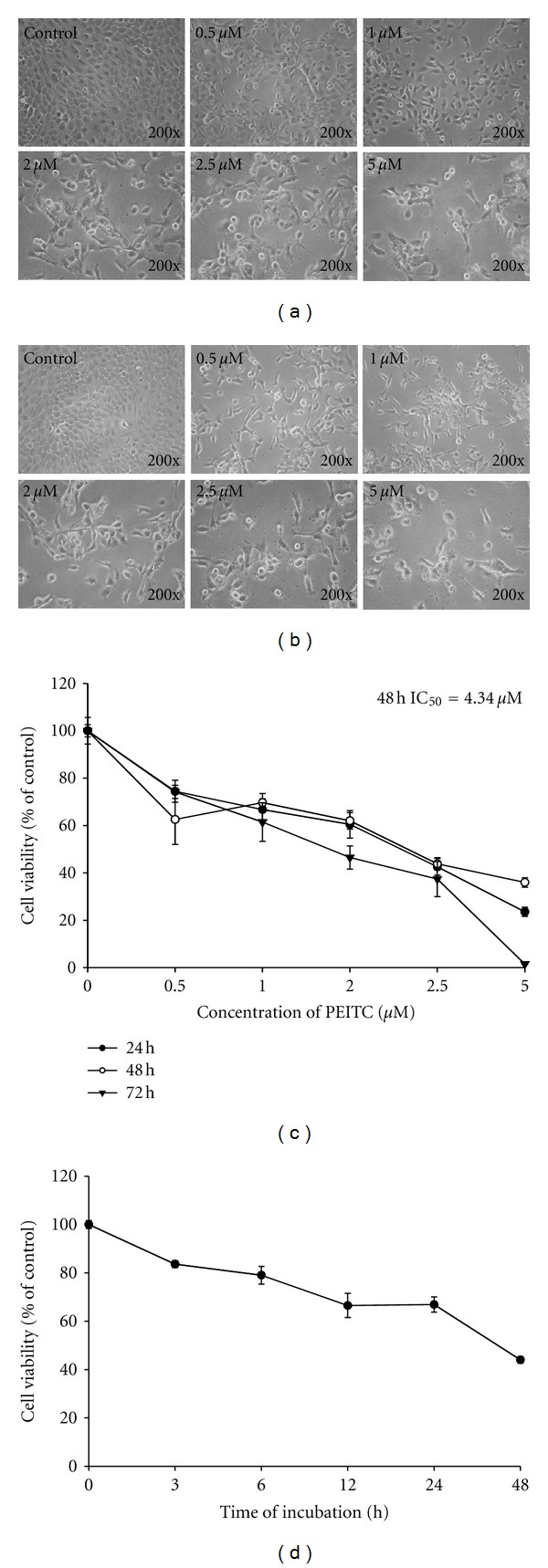
PEITC affected on cell viability and morphological changes in HSC-3 cells. Cells were treated with 0.5, 1, 2, 2.5 and 5 *μ*M of PEITC for 24 and 48 h or were treated with 2.5 *μ*M of PEITC for 0, 3, 6, 12, 24, and 48 h. DMSO at 0.5% is as a vehicle control. Cell morphological changes were examined and photographed under contrast phase microscope (a: 24 h; b: 48 h). The percentage of viable cells was measured by flow cytometric assay (c and d). Data are presented as the mean ± S.E.M. of three independent experiments. *P < 0.05, significantly different compared with control treatment.

To evaluate the effect of the PEITC on cell-morphological changes and the viability of HSC-3 cells, we treated HSC-3 cells with various concentrations (0.5, 1, 2, 2.5, and 5 *μ*M) for 24, 48, or 72 h. The cell-morphological changes were examined and photographed by a phase-contrast microscope. The results shown in Figures 1(a) and 1(b) indicated that PEITC induced cell-morphological changes, cell body shrinkage, and cell number reduction for 24 and 48 h after the exposure to 0.5–5 *μ*M of PEITC. Furthermore, the percentage of viable cells was measured by flow cytometric assay. Then cells were treated with various doses of PEITC for 24, 48, and 72 h, and PEITC decreased the percentage of viable cells in a dose- and time-dependent manner (Figures 1(c) and 1(d)).

### 3.2. PEITC Induced *G*_0_/*G*_1_ Phase Arrest in HSC-3 Cells

**Figure 2:**
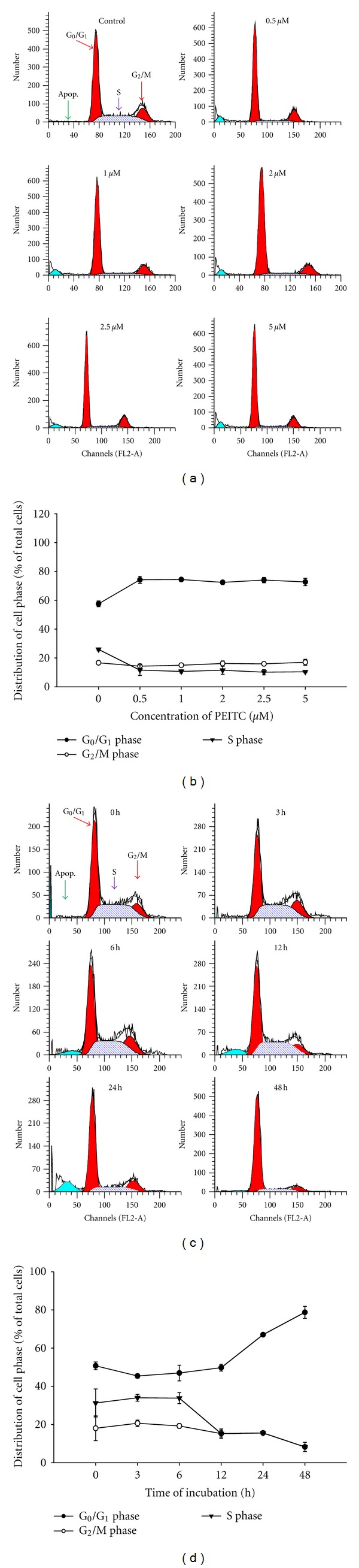
PEITC affected on cell-cycle distribution in HSC-3 cells. Cells were treated with 0, 0.5, 1, 2, 2.5, and 5 *μ*M of PEITC for 24 h or were treated with 2.5 *μ*M of PEITC for 0, 3, 6, 12, 24, and 48 h. The cell-cycle distribution was determined by using flow cytometric analysis (a and c), and cell-cycle distribution was quantified (b and d). Data are presented as the mean ± S.E.M. of three independent experiments. *P < 0.05, significantly different compared with 0 h treatment.

In order to determine whether PEITC could block the cell cycle progression of HSC-3 cells, the cell-cycle distribution of PEITC-treated cells was evaluated by flow cytometric analysis, and results are shown in Figure 2. As a consequence of PEITC treatment, the percentage of HSC-3 cells in the *G*_0_/*G*_1_ phase increased from 50% in the controls to 75% at 0.5 *μ*M and 75% at 5 *μ*M, with a concurrent decline in the G_2_ and S phase, in a dose-related fashion (Figures 2(a) and 2(b)). Furthermore, HSC-3 cells were treated with 2.5 *μ*M for 0, 3, 6, 12, 24, and 48 h, and then cells were arrested at *G*_0_/*G*_1_ phase in a time-dependent manner (Figures 2(c) and 2(d)). These results show that PEITC arrested cell cycle at *G*_0_/*G*_1_ phase in the HSC-3 cells.

### 3.3. PEITC Induced Degrades Nuclei in HSC-3 Cells

**Figure 3:**
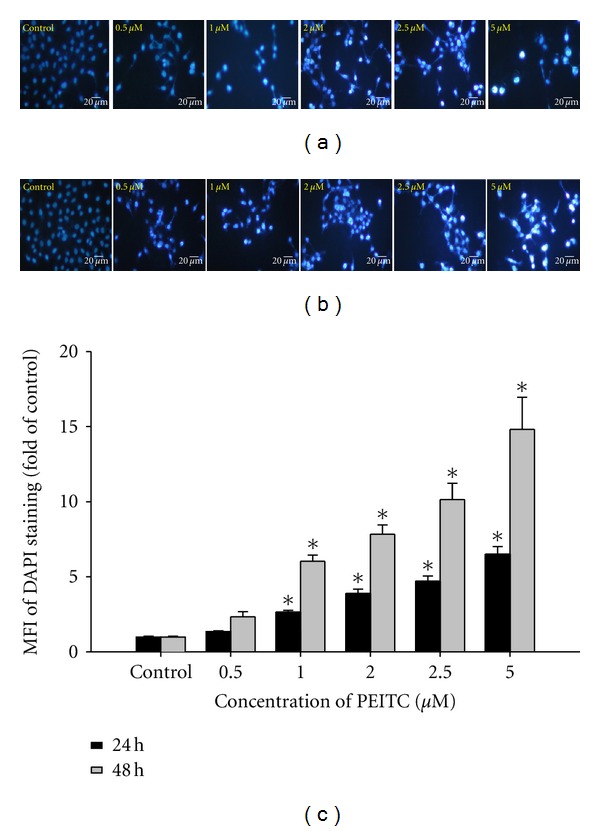
PEITC induced cell apoptosis in HSC-3 cells. Cells were treated with 0, 0.5, 1, 2, 2.5, and 5 *μ*M of PEITC for 24 and 48 h. DAPI staining was determined by immunostaining and photographed by fluorescence microscopic systems (200x) as described in Materials and Methods (a: 24 h; b: 48 h). Scale bar = 20 *μ*m. The MFI of DAPI staining was calculated (c). Data are presented as the mean ± S.E.M. of three independent experiments. *P < 0.05, significantly different compared with PEITC-treatment.

To confirm that prolonged incubation with PEITC-induced apoptosis in HSC-3 cells, an additional apoptotic marker, DAPI nuclear staining was evaluated, and results are shown in Figure 3. The Figures 3(a) and 3(b) show that the apoptotic bodies with condensed chromatin and degraded nuclei were observed after DAPI staining, and this effect is a dose-dependent manner. After calculated from Figures 3(a) and 3(b), the data shown in Figure 3(c) indicated that degraded nuclei were increased when the doses of PEITC and time treatment were increased.

### 3.4. PEITC Affected Reactive Oxygen Species (ROS) Production, Levels of ΔΨ_*m*_ and Cytosolic Ca^2+^ release in HSC-3 Cells

**Figure 4:**
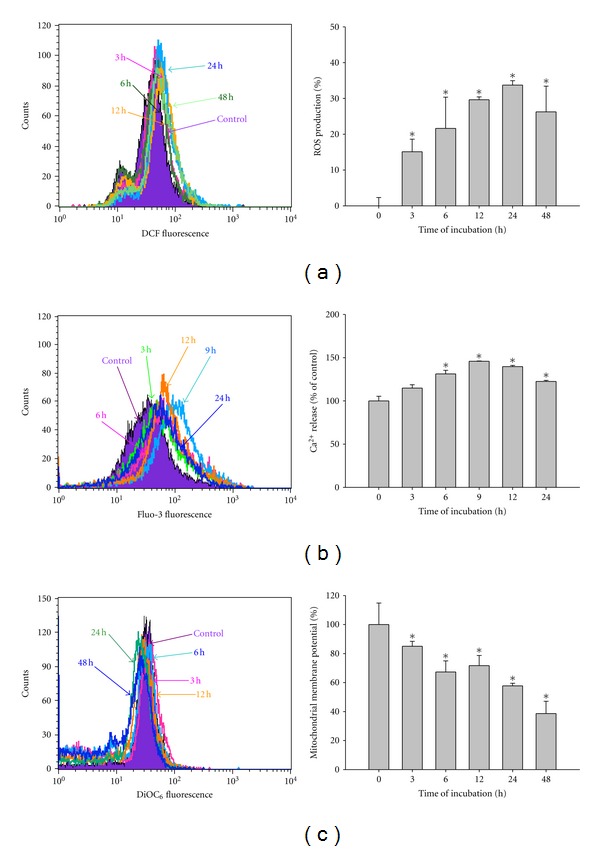
PEITC affected the reactive oxygen species (ROS) productions, intracellular Ca^2+^ release, and the levels of mitochondrial membrane potential (ΔΨ_*m*_) in HSC-3 cells. Cells were treated with 2.5 *μ*M of PEITC for 0, 3, 6, 9, 12, 24, and 48 h for the production of ROS (a) and Ca^2+^ (b), or incubation for 0, 3, 6, 12, 24, and 48 h in the changes in ΔΨ_*m*_ (c). All samples were analyzed by flow cytometric assay as described in Section 2. Data are presented as the mean ± S.E.M. of three independent experiments. *P < 0.05, significantly different compared with PEITC-treatment.

We confirmed that whether PEITC-induced apoptosis is accompanied by the production of ROS and Ca^2+^ and also to investigate the role of mitochondria in PEITC-triggered cell death. The results are shown in Figures 4(a), 4(b) and 4(c), which indicated that PEITC promoted the production of ROS (Figure 4(a)) and Ca^2+^ (Figure 4(c)) but decreased the levels of ΔΨ_*m*_ (Figure 4(b)) in a time-responded manner.

### 3.5. PEITC Altered the Protein Levels Associated With *G*_0_/*G*_1_ Phase Arrest and Apoptosis in HSC-3 Cells

**Figure 5:**
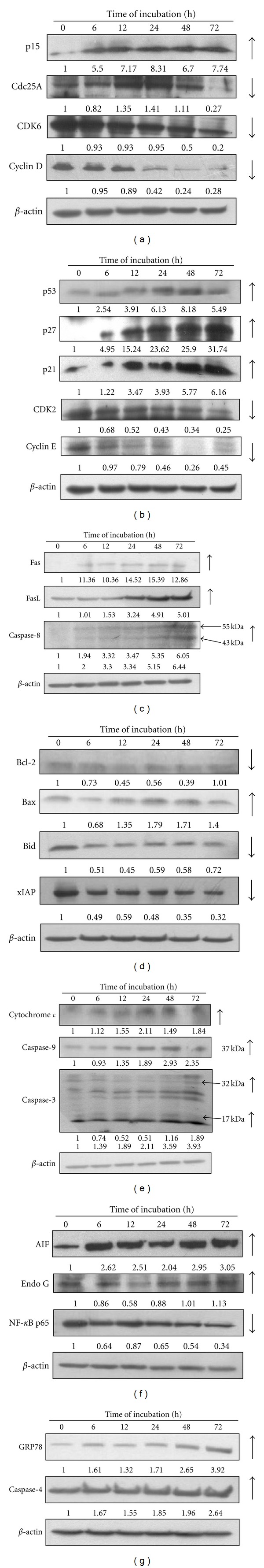
PEITC altered the levels of *G*_0_/*G*_1_ phase and apoptotic relative proteins in HSC-3 cells. Cells were exposed to PEITC (2.5 *μ*M) and then incubated for 0, 6, 12 24, 48, and 72 h. Then cells were harvested for total protein determination then the protein levels of p15, cdc25A, CDK6, and cyclin D (a), p53, p27, p21, CDK2, and cyclin E (b), Fas, FasL, and caspase 8 (c), Bcl-2, Bax, Bid, and xIAP (d), cytochrome *c*, caspase 9, and 3 (e), AIF, Endo G, and NF-*κ*B p65 (f), GRP78 and caspase 4 (g) were determined protein levels by Western blotting, and then protein levels were quantified by NIH ImageJ software. Data are presented from three independent experiments with similar results.

To explore the mechanisms underlying PEITC-induced *G*_0_/*G*_1_ phase arrest and apoptosis, the levels of *G*_0_/*G*_1_ and apoptosis-regulated proteins in HSC-3 cells after exposure to 2.5 *μ*M of PEITC for 0, 6, 12, 24, 48, and 72 h were evaluated. The results shown in Figure 5 from Western blot analysis displayed that PEITC treatment resulted in substantial reductions in the levels of cdc25A, CDK6, and cyclin D (Figure 5(a)), CDK2 and cyclin E (Figure 5(b)) proteins, but increased that of p15 (Figure 5(a)), p53, p27, and p21 (Figure 5(b)) that led to *G*_0_/*G*_1_phase arrest. Furthermore, PEITC promoted the expression of apoptosis-related protein levels such as Fas, FasL, and caspase-8 (Figure 5(c)), Bax (Figure 5(d)), cytochrome *c*, caspase-9 and -3 (Figure 5(e)), AIF and Endo G (Figure 5(f)), GRP 78, and caspase-4 (Figure 5(g)) but decreased the levels of Bcl-2, Bid and xIAP (Figure 5(d)) and NF-*κ*B p65 (Figure 5(f)) that led to cell apoptosis in HSC-3 cells.

### 3.6. The PEITC Binding Site

**Figure 6:**
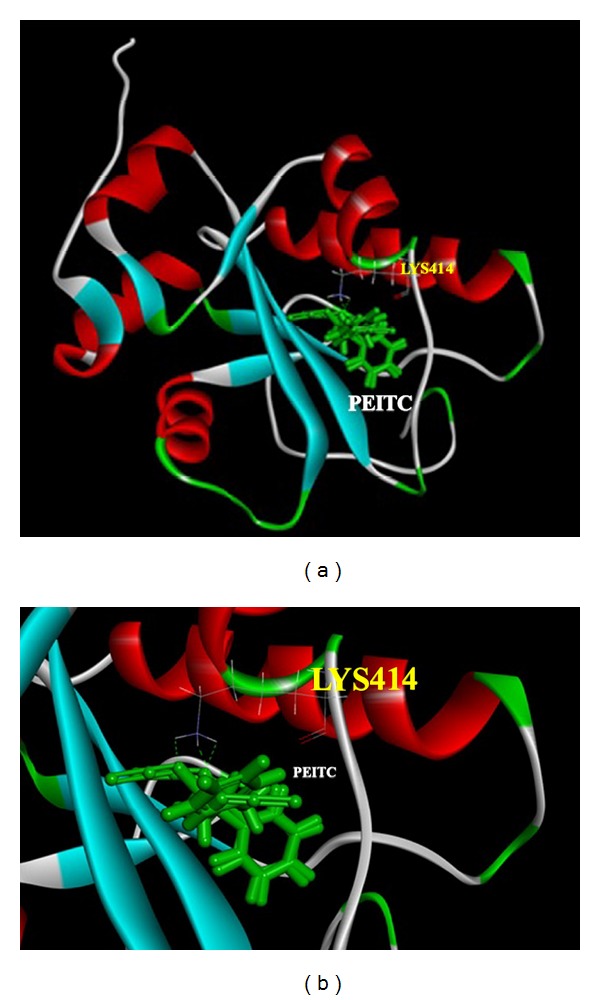
The conformation of PEITC (ligand) in the cdc25A (protein) in the binding site and the three hydrogen bond interactions that are between the PEITC and cdc25A.

The active site is located in the center of the cdc25A protein, as Figures 6(a) and 6(b) illustrated. It shows that the PEITC is directly perpendicular to the LYS414 and form three hydrogen bonds. The structure of PEITC contains two major parts: the aromatic ring (hydrophobic part) and the cyanate (hydrophilic part). The cyanate (hydrophilic part) will form three hydrogen bonds directly with LYS414, which contain OH group and NH groups.

## 4. Discussion

Other investigators already pointed out that ROS might be fully involved in cellular responses to PEITC [31, 32], and our previous studies have confirmed that PEITC promoted ROS production which associated with the induction of apoptosis in human cancer cells [19, 20]. Herein, we also showed that PEITC induced cytotoxic effects on HSC-3 cells through the induction of apoptosis, and it also related to the involvement of ROS* via *mitochondria-dependent signal pathways. This suggestion was based on the observations: (1) the PEITC treatment decreased the percentage of viable cells and induced cell morphological changes in a dose-dependent manner; (2) PEITC induced apoptosis in a time-dependent manner; (3) PEITC promoted the production of ROS and Ca^2+^ but decreased the levels of ΔΨ_*m*_ in a time-dependent manners; (4) PEITC increased the proapoptotic protein Bax and decreased the antiapoptotic protein Bcl-2, both proteins involved the levels of ΔΨ_*m*_ for cell to survive or apoptosis [33].

Furthermore, our results also show that PEITC decreased expression of cdc25A, CDK6 and cyclin D (Figure 5(a)), CDK2 and cyclin E (Figure 5(b)) proteins but increased the levels of p15 (Figure 5(a)), p53, p27, and p21 (Figure 5(b)) that led to *G*_0_/*G*_1_ phase arrest in HSC-3 cells. Thus, PEITC induced downregulation of cyclins and CDKs and upregulation of the CDK inhibitor p21 expression, whereas upregulation of p27 for causing *G*_0_/*G*_1_ arrest. In this regard, direct covalent modification of cellular proteins has been suggested to be an important early event in the induction of cell-cycle arrest by PEITC [19]. It is well documented that CDKs activity is governed by regulatory subunits (cyclins) with their catalytic subunit CDKs to form complex and are regulated at a specific phase of the cell cycle [34–36]. The cyclin/CDK complexes regulated the cells from transit through G_1_ of the cell cycle and entry into the S phase, which are predominantly cyclin D1/CDK4 and cyclin E/CDK2 [34]; moreover, the kinase activities of cyclin/CDK complexes are negatively regulated by CDK inhibitors, such as p21 and p27 [34, 37]. However, the other report demonstrated that PEITC induced G_2_/M arrest in PC-3 human prostate cancer cells [38] and HL-60 human leukemia cells [39]. It has been reported that PEITC-induced G_2_/M arrest was associated with proteasome-mediated degradation of CDK1 and cdc25c [39]. However, it was also reported that PEITC induced *G*_0_/*G*_1_ phase arrest in HT29 human colon cancer cells [40]. In addition to *G*_0_/*G*_1_ phase arrest, we found that PEITC treatment caused a decrease of S-phase cells in HSC-3 cells. Thus, the different cell lines which may have different responses are not clear at present study. Apparently, further investigations are needed.

It was reported that ROS had function upstream of cytochrome *c* release and caspase-3 activation by certain apoptotic stimuli such as hyperoxia [19] and the generation of ROS downstream of the release of cytochrome *c* in some cellular models of mitochondria-mediated apoptosis [41]. Here, we found that PEITC promoted ROS production and decreased the levels of ΔΨ_*m*_ and cytochrome *c* release, the activation of caspase-9 and caspase-3 (Figure 6(e)) for causing apoptosis or through AIF and Endo G release (Figure 6(f)), leading to apoptosis. The present study also demonstrates that PEITC treatment causes ROS-dependent activation (Figure 4(a)) and mitochondrial translocation of Bax (Figure 5(d)). Hydrogen bonds are a type of dipole-dipole interaction formed between the proton of a group X-H, where X is an electronegative atom, and other electronegative atoms (Y) containing a pair of nonbonded electrons. The hydrogen bond (5 to 30 kJ/mole) is stronger than a van der Waals interaction, but weaker than covalent or ionic bonds. The hydrogen bonds become important in intermolecular bonding between the PEITC and the cdc25A (Figures 6(a) and 6(b)).

**Figure 7:**
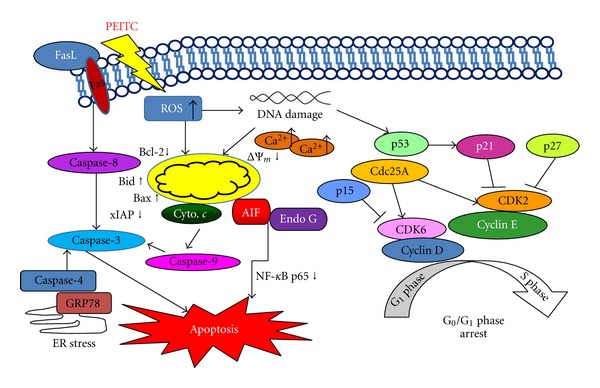
The proposed model of PEITC mechanisms of action for *G*_0_/*G*_1_ phase arrest and apoptotic cell death in human oral squamous carcinoma HSC-3 cells.

In conclusion, PEITC induced apoptosis in HSC-3 cells which are summarized in Figure 7. We suggest that PEITC might be through Fas and FasL, promotion of ROS and Ca^2+^ production that caused ER stress which based on increasing the GRP78 and ROS, then led to the changes of Bax/Bcl-2 for causing the decreased of ΔΨ_*m*_ and then led to cytochrome *c*, AIF and Endo G release, then to activate caspase-9 and -3 for causing apoptosis.
